# Protective effect of soursop (*Annona muricata* linn.) juice on oxidative stress in heat stressed rabbits

**DOI:** 10.1186/s40781-018-0186-4

**Published:** 2018-11-16

**Authors:** Olatunji Abubakar Jimoh, Eyanlola Soladoye Ayedun, Waheed Abimbola Oyelade, Olugbenga David Oloruntola, Olajumoke Temidayo Daramola, Simeon Olugbemiga Ayodele, Idowu Samuel Omoniyi

**Affiliations:** 1 Department of Agricultural Technology, Federal Polytechnic Ado Ekiti, Ado Ekiti, Ekiti State Nigeria; 2 Department of Science Technology, Federal Polytechnic, Ado Ekiti, Ekiti State Nigeria; 3 Department of Animal Science, Adekunle Ajasin University Akungba Akoko, Akungba Akoko, Ondo State Nigeria

**Keywords:** Antioxidants, Glutathione, Heat stress, Lipid peroxide, Rabbit, Soursop

## Abstract

**Background:**

Preventing oxidative stress in heat stressed animals may be possible by increasing antioxidant defence via exogenous administration of antioxidant substrate and/or its precursors. The study aimed to investigate the effect of Soursop juice in mitigating oxidative stress induced by heat stress in rabbit.

**Methods:**

Sixty mixed breed rabbit bucks aged 12–18 months old with the average weight of 1826 ± 8.35 g/rabbit, randomly allotted to experimental treatments of four replicates each, in a completely randomized design during high-temperature humidity index in Ado Ekiti, Southwest Nigeria. Soursop juice (SSJ) was administered via oral drenched daily per kg body weight (BW), to designated treatment 1 to 5; 0.55 mlkg^−1^BW distilled water (control), 0.55 mlkg^−1^BW SSJ, 1.11 mlkg^−1^BW SSJ, 1.67 mlkg^−1^BW SSJ and 2.22 mlkg^−1^BW SSJ, respectively. Fastened blood samples were collected at days 28 and 56, and assay for serum protein, cholesterol, triglycerides, superoxide dismutase, catalase, reduced glutathione and lipid peroxidation using standard procedures.

**Result:**

Result revealed that SSJ demonstrated hypocholesterolemic effect in a dose-dependent manner throughout the study. Effect of chronic administration of SSJ to heat stressed rabbits proved beneficial, as SSJ reduced serum lipid peroxidation and enhanced antioxidant activity over 8 weeks.

**Conclusion:**

Administration of soursop juice to heat-stressed bucks at 2.22 mlkg^−1^BW offered optimum antioxidant defense against oxidative stress.

## Background

Heat stress is one of the most important stressors especially in hot regions of the world. Effects of heat stress on rabbits bucks has been widely documented to include reduced productivity, compromised the immune system, collapse inefficient thermoregulatory mechanism and reduce fertility when environmental variables induce heat stress [1]. Most recently, exotic breeds of rabbit were introduced to Nigeria, to improve rabbit population in the region. It was observed that oxidative stress greatly compromised animal physiology, with high reactive oxygen species and its metabolites (ROS-M) accumulation in blood and seminal fluid of bucks during heat stress [2]. The accumulations of ROS-M in the biological system is a resultant of decline in antioxidant defense system, notably enzymatic antioxidants and total antioxidant activity. The endogenous production of antioxidant enzymes and the total antioxidant activity of rabbit bucks decline in huge proportion when animals experience heat stress compared to when the environmental variables depict the absence of heat stress [3].

Jimoh et al.*,* [2] reported that mitigating strategies to ameliorate the adverse effects of heat stress in rabbits are imperative for productivity. The possibility of increasing antioxidant defence through exogenous administration of antioxidant substrate and/or its precursors requires investigation. Soursop (*Annona muricata*.L.) belongs to the family Annonaceae which is found throughout the tropics. Several studies have described the medicinal purposes of *Annona muricata* fruit, leaf, bark, and seed; sedative, antispasmodic, hypoglycemic, hypotensive, smooth muscles relaxant and nervine, cytotoxic against cancer cells. Soursop possess protective and beneficial effect against oxidative stress in rats by decreasing lipid peroxidation and enhancing endogenous antioxidants [4]. These prove as a potential candidate to mitigate oxidative stress induced by heat stress in the rabbit in the tropics. This study investigates the protective effect of soursop juice against heat stress-induced oxidative stress in rabbit bucks.

## Methods

The study was conducted at the rabbit unit of Teaching and Research Farm, Department of Agricultural Technology, Federal Polytechnic, Ado-Ekiti, Nigeria. The study area is located between latitude 7^o^3′7″N 7′12″N and latitude 5^o^11″E and 5^o^ 31″E. The temperature-humidity index (THI), an indicator of thermal comfort level for animals in an enclosure was calculated as modified by Marai et al. [5] and given as:

$$ \mathrm{THI}=\mathrm{t}-\Big[\left(0.31-0.31\ \mathrm{x}\ \mathrm{RH}\ \mathrm{t}-14.4\right) $$

Where RH = relative humidity /100. t = ambient temperature.

The values of THI obtained for rabbit are classified as:

< 27.8 °C = absence of heat stress,

27.8–28.9 °C = moderate heat stress,

28.9–30 °C = severe heat stress and.

above 30 °C = very severe heat stress (Marai et al.,) [5].

The study was conducted between December 2017 and January 2018 with average daily THI of 29.94 ± 1.56 and 28.59 ± 3.10 at the rabbit unit, which is the peak of the dry season in the study area at the time.

### Collection and extraction of soursop juice

Ripe soursop (*Annona muricata*) fruit was harvested fresh and it was extracted in the Teaching and Research Farm of the Agricultural Technology, Federal Polytechnic Ado –Ekiti, Nigeria. The fruits were peeled, weighed and the fruit pulp blended (without the seeds) with the ratio of 1:2 (pulp: distilled water). The soursop juice was clarified with juice extractor (Mikachi model No 1706). The juice obtained was designated as soursop juice (SSJ) and kept frozen 4 °C in disposable 5 ml sterile sample bottles until its required for use.

### Experimental animal and management

Sixty (60) mixed breed (New Zealand white x Chinchilla) male rabbit bucks kept in a battery cage system kept in an open-sided house were used for the experiment. The rabbit house has natural lighting program (12D:12 L), with the dynamic ventilation system. The experimental units are made of wire mesh double sided boxes of 1.1sq.m. The animals were fed ad libitum with diets containing crude protein 17.05%, digestible energy 2592.06 Kcal/kg, crude fibre 10.02%, calcium 0.45% and phosphorus 0.21% as shown in Table 1. Freshwater was made available to the animals always. Other routines and periodic management practices necessary for rabbit production were carried out.

**Table 1:** Gross composition of experimental diet

Ingredient	Inclusion (g/100 g)
Maize	25
Wheat offal	5
Rice bran	6
BDG	5
SBM	17
Groundnut haulms	40
Methionine	0.4
Lysine	0.1
Bone meal	1
Premix	0.25
Salt	0.25
Total	100
Calculated nutrient composition	
Dry matter (%)	87.87
Crude protein (%)	16.44
Digestible energy (kcal/kg)	2717.86
Ether extract (%)	3.34
Crude fibre (%)	16.48
Lysine (%)	1.01
Methionine (%)	0.75
Calcium (%)	1.60
Phosphorus (%)	0.45

The bucks were adult of 12-18 months old with the average weight of 1826 ± 8.35 g. 60 bucks were randomly allotted to five experimental treatments of four replicates (3 bucks per replicate) per treatment in a completely randomised design. SSJ was administered per kg body weight (BW), designated as treatment 1 to 5, administered 0.55mlkg^−1^BW distilled water (control), 0.55 mlkg^−1^BW SSJ, 1.11 mlkg^−1^BW SSJ, 1.67 mlkg^−1^BW SSJ and 2.22 mlkg^−1^BW SSJ respectively. Each treatment consists of 12 bucks. The soursop juice was administered orally to the rabbit in the morning between 8:00 am and 8:30 am daily to all bucks in a study that lasted for eight weeks.

### Sample collection and analysis

A baseline blood sample was collected from all rabbits at least temperature humidity index, to serve as positive control as non-heat stressed treatment. The fastened blood sample was collected at 28 days and 56 days of the study. Blood was collected through the marginal ear vein into a sample bottle. Serum was separated by centrifugation (Medi Scan centrifuge model No 800D) and stored at -2 °C before analysis. Serum obtained was assayed for total protein, albumin, cholesterol, and triglyceride using commercial assay kits and its procedure, lipid peroxidation, reduced glutathione, catalase, and superoxide dismutase activity using standard procedures.

Superoxide dismutase (SOD); to 2.1 ml of 50 m M butter, 0.02 ml of enzyme source and 0.86 ml of distilled water. The reaction is initiated with 0.02 ml of 10 mM pyrogallol and change in absorbance monitored at 420 mm. One unit of SOD is defined as that amount of enzyme required to inhibit to auto-oxidation of pyrogallol by 50℅ in standard assay system of 3 ml. The specific activity is expressed as unit /min/mg protein.

Catalase activity; the assay system contain 1.9 ml,0.05 M buffer PH 7.0 and 1.0 ml 0.059 M water. The reaction is initiated by addition of 0.1 ml enzyme source. The decrease in absorbance is monitored at 1 min interval for 5 min at 240 min and activity is expressed as moles of water decomposed /min/mg protein.

The assay for lipid peroxidation; The reaction mixture in a total volume of 3.0 ml contained 1.0 ml serum, 1.0 ml of TCA (0.67%). All the test tubes were placed in a boiling water bath for a period of 45 min. The tubes were shifted to the ice bath and then centrifuged at 2500 rpm for 10 min. The amount of malondialdehyde (MDA) formed in each of the samples was assessed by measuring the optical density of the supernatant at 532 nm.

The glutathione (GSH) content; in which 1.0 ml of PMS fraction (10%) was mixed with 1.0 ml of sulphosalicylic acid (4%).The sample was incubated at 4 °C for at least 1 h and then subjected to centrifugation at 1200 rpm for 15 min at 4 °C. The assay mixture contained 0.4 ml filtered aliquot, 2.2 ml phosphate, buffer (0.1 M, PH 7.4) and 0.4 ml DTNB (10 mM) in a total volume of 3.0 ml. The yellow colour developed was read immediately at 412 mm.

### Statistical analysis

Data obtained were subjected to analysis of variance at α = 0.05, the general linear model procedure of SAS, while means were separated using Duncan’s multiple range test of same software.

The statistical model is as the following:

$$ {\mathrm{Y}}_{ijl}=\upmu +{\mathrm{B}}_i+{\mathrm{e}}_{ijl} $$

Where Y_*ijl*_ represents the value of serum biochemical parameters and oxidative status measured in the *l*^th^ animal; μ is the overall mean for each character; B_*i*_ is the fixed effect of *i*^th^ Soursop (SSJ) was administered 5 levels (*i* = 0.55 mlkg^−1^BW water (control), 0.55 mlkg^−1^BW SSJ, 1.11 mlkg^−1^BW SSJ, 1.67 mlkg^−1^BW SSJ and 2.22 mlkg^−1^BW SSJ); and e_*ijl*_ is the random residual effect.

## Results

Serum oxidative status of non-heat stressed treatment is shown in Table 2. Total protein, antioxidant activities were higher than values obtained in heat stressed groups. However, cholesterol and lipid peroxidation were lower in non-heat stressed group compared with heat stressed groups.

**Table 2:** baseline of serum oxidative status of the experimental rabbits at absence of heat stress

Parameters	Baseline assessment (un-heat stressed treatment)
Total Protein (g/dL)	54.8
Cholesterol (mg/dL)	51.04
Triglyceride (mg/dL)	114.53
Lipid Peroxidation (TBARS/mg protein)	0.09
Glutathione (μg GSH /min/mg protein)	54.05
Catalase (nm H_2_O_2_ /min/mg protein)	132.77
Superoxide Dismutase (U/min/mg protein)	78.68

### Serum oxidative status of heat-stressed bucks gavaged with soursop juice

Serum oxidative status of heat stressed bucks gavaged with soursop juice for 4 weeks is shown in Table 3. The serum total protein of bucks on control were statistically (*p* > 0.05) similar with bucks on treatments 3, 4 and 5. Cholesterol of bucks on SSJ based treatments were significantly (*p* < 0.05) lower than those on treatment 1. Triglyceride declined with increased SSJ administration. Bucks on treatments 2, 3, 4 and 5 had significantly (*p* < 0.05) low values while bucks not offered SSJ had the statistically highest value. Serum lipid peroxidation was significantly (*p* < 0.05) highest in the buck on control and least in bucks offered treatments 4 and 5. However, bucks on treatments 2 and 3 had statistically (*p* > 0.05) similar lipid peroxidation values and were significantly (p < 0.05) higher than bucks on treatments 4 and 5. Glutathione of bucks on treatments 4 and 5 was significantly (*p* < 0.05) highest across the treatments with significantly least values obtained in bucks on treatments 1 and 2. Superoxide dismutase activity was highest statistically in bucks on treatment 5, with bucks on treatments 1 and 2 having the significantly (p < 0.05) least values. Bucks on treatment 3 and 4 had lower SOD than bucks on treatment 5. Serum catalase activity in SSJ based groups was significantly higher than the control.

**Table 3:** Serum oxidative status of heat stressed bucks gavaged with soursop juice for 4 weeks

		Treatment 1 (0 mlkg^− 1^ BW)	Treatment 2 (0.55 mlkg^− 1^ BW)	Treatment 3 (1.11 mlkg^− 1^ BW)	Treatment 4 (1.67 mlkg^− 1^ BW)	Treatment 5 (2.22 mlkg^−1^ BW)	SEM
Total Protein (g/dL)	54.8	14.92^a^	8.57^b^	14.28^a^	13.18^a^	11.10^a^	1.24
Cholesterol (mg/dL)	51.04	61.17^a^	48.26^b^	46.95^b^	50.47^b^	50.88^b^	3.86
Triglyceride (mg/dL)	114.53	148.01^a^	129.61^b^	103.49^c^	103.31^c^	99.52^c^	9.09
Lipid Peroxidation (TBARS/mg protein)	0.09	1.46^a^	0.90^b^	0.96^b^	0.79^c^	0.75^c^	0.05
Glutathione (μg GSH /min/mg protein)	54.05	17.39^c^	20.90^c^	27.08^b^	33.86^a^	38.46^a^	3.48
Catalase (nm H_2_O_2_ /min/mg protein)	132.77	54.01^b^	95.05^a^	87.38^a^	90.02^a^	96.50^a^	6.38
Superoxide Dismutase (U/min/mg protein)	78.68	21.74^c^	24.64^c^	35.87^b^	38.26^b^	60.63^a^	3.62

Different superscipt denote statistically significant differences at *p ≤ 0.05*

Serum oxidative status of heat stressed bucks gavaged with soursop juice for 8 weeks (Table 4) reveal that serum total protein in bucks on treatments 3, 4 and 5 are statistically similar and significantly (*p* < 0.05) lower than bucks on treatments 1 and 2. Serum cholesterol of bucks on treatments 1, 2 and 3 are similar and were statistically (*p* < 0.05) higher than bucks on treatment 4 and 5. Triglyceride of bucks on control was significantly (*p* < 0.05) higher than bucks on SSJ based treatments. Serum lipid peroxidation of bucks on treatment 1 was significantly (p < 0.05) highest, while bucks on treatment 5 had significantly (*p* < 0.05) least values. Serum glutathione activity of bucks on SSJ treatments were statistically similar and significantly (p < 0.05) higher than bucks on control. Serum catalase activity in SSJ based groups was significantly higher than the control. Superoxide dismutase activity of bucks on treatment 1, 2, 3 and 4 was statistically similar and significantly (p < 0.05) lower than SOD of buck on treatment 5.

**Table 4:** Serum oxidative status of heat stressed bucks gavaged with soursop juice for 8 weeks

	Baseline assessment (un-heat stressed treatment)	Treatment 1 (0 mlkg^−1^ BW)	Treatment 2 (0.55 mlkg^−1^ BW)	Treatment 3 (1.11 mlkg^− 1^ BW)	Treatment 4 (1.67 mlkg^− 1^ BW)	Treatment 5 (2.22 mlkg^− 1^ BW)	SEM
Total Protein (g/dL)	54.8	43.39^a^	39.20^a^	32.70^b^	29.60^b^	24.15^b^	2.27
Cholesterol (mg/dL)	51.04	57.91^a^	50.19^a^	51.91^a^	41.03^b^	36.68^b^	2.97
Triglyceride (mg/dL)	114.53	214.78^a^	134.15^b^	177.05^b^	155.75^b^	129.77^b^	10.89
Lipid Peroxidation (TBARS/mg protein)	0.09	1.41^a^	0.35^b^	0.38^b^	0.29^b^	0.19^c^	0.04
Glutathione (μg GSH /min/mg protein)	54.05	27.79^b^	37.28^a^	38.01^a^	45.31^a^	45.99^a^	4.29
Catalase (nm H_2_O_2_ /min/mg protein)	132.77	43.58^b^	102.42^a^	100.89^a^	103.36^a^	109.03^a^	7.29
Superoxide Dismutase (U/min/mg protein)	78.68	17.39^b^	20.29^b^	19.57^b^	21.74^b^	28.70^a^	3.01

Different superscipt denote statistically significant differences at *p ≤ 0.05*

## Discussion

Cholesterol and triglyceride values of SSJ drenched bucks were lower than the control over 8 weeks. This shows that SSJ demonstrated the hypocholesterolemic effect in a dose-dependent manner throughout the study, as evident in cholesterol and triglyceride values. This corroborates reports that *A. muricata* lowers hypertriglyceridemia and hypercholesterolemia in alloxan-induced diabetic rats [6]. Zheng et al. [7] demonstrated that fruit and vegetable juice reduce total cholesterol in the body and improve cholesterol profile in the blood. The improving blood lipids effects of the juice blend were attributed to the high content of total polyphenol. The trend of result shows that total protein of SSJ drenched bucks was lower than control and increase across the treatments with the period of administration.

SSJ reduced serum lipid peroxidation throughout the study, this proves a beneficial effect of chronic administration of SSJ on heat stressed bucks. This is revealed by values of lipid peroxidation in SSJ drenched bucks, which were lower at 8 weeks than at 4 weeks of the study. This reveals that the duration of administration of SSJ enhanced mitigation of oxidant accumulations and/or production in heat stressed bucks. Hypercholesterolemia is related to increased lipid peroxidation [7], thus the mechanism of soursop juice reduction of cholesterol contributes to lowering lipid peroxidation as revealed in this work. Similarly, cranberry powder has been shown to decrease the accumulation of malondialdehyde (MDA, an indicator of oxidative damage) in male New Zealand rabbit kidneys [8]. This is also supported by claims that *A. muricata* extract has a protective and beneficial effect on hepatic tissues subjected to STZ-induced oxidative stress, by decreasing lipid peroxidation and indirectly enhancing production of insulin and endogenous antioxidants [4].

Glutathione activity in heat stressed bucks increased with the dosage of SSJ administration at 4 weeks. However, at 8 weeks though values were higher than at 4 weeks, the effect of the accumulation of production of glutathione could account for their similar values in SSJ drenched groups. The least values of glutathione were obtained in control group throughout the study, this demonstrates the enhancement of glutathione activity in heat stressed bucks by SSJ to counteract the accumulation of hydrogen peroxide. This is also observed in catalase activity of the bucks, catalase and glutathione are responsible for the elimination of hydrogen peroxide in biological systems. Similarly, ginger also a natural antioxidant spice has been reported to decrease lipid peroxidation, increasing GSH content and maintaining normal levels of antioxidant enzymes in rats [9]. Effect of chronic antioxidant-rich fruit administration was also reported by Kota et al. [10] in rat supplemented ginger for one month, which recorded significant increases in activity of antioxidant enzymes (superoxide dismutase, glutathione peroxidase, and catalase) in the liver. Superoxide dismutase activity was higher at 4 weeks in SSJ drenched bucks than at 8 weeks. This could be due to the first line defense of SOD to convert superoxide anion to hydrogen peroxide in heat stressed bucks at 4 weeks. After regulating superoxide anion production and elimination, SOD activity declines to favour catalase and glutathione activity at the latter stage, to counteract the hydrogen peroxide being produced as a metabolite of SOD. It suggests that SOD functions in tandem with catalase and glutathione regulate superoxide anion and hydrogen peroxide productions and/or accumulation in the bucks. This result in mitigating oxidants (lipid peroxides) and prevent oxidative stress in heat stressed bucks. This demonstrates SSJ mechanism to mitigate oxidative stress in heat stressed bucks. Contrariwise, cranberry and ginger had no effect on the oxidative stress or antioxidant status in exercising horses [11]. This study also agrees with the notion that antioxidant-rich juice/extracts are more effective when the body has undergone intense, long-term oxidative stress, rather than in healthy individuals or in individuals undergoing short-term or acute oxidative stress [11]. Zheng et al. [7] review antioxidant effects of several fruits and vegetable juice and concluded that the consumption of juice enhanced total antioxidant activity and enzymatic antioxidants and are important therapies to ameliorate oxidative stress and its related diseases. Similarly, Syahida et al. [12] reported that *Annona muricata* L. extract contains the high total antioxidant, which improved the total antioxidant status of Sprague-Dawley rats as the dosage increased, which is good in promoting health.

## Conclusion

This study reveals that chronic oral administration of soursop juice proved beneficial in promoting good health of heat-stressed bucks. Soursop juice reduced serum lipid peroxidation and enhanced antioxidant activity in heat stressed rabbit over 8 weeks. Administration of soursop juice to heat-stressed bucks at 2.22 mlkg^−1^BW offered optimum antioxidant defense against oxidative stress.

## Abbreviations

BW: Body weight
GSH: Glutathione
MDA: Malondialdehyde
ROS-M: Reactive oxygen species and its metabolites
SAS: Statistical analysis software
SOD: Superoxide dismutase
SSJ: Soursop juice
THI: Temperature-humidity index
